# Evaluation of global and regional myocardial work by echocardiography in patients with Fabry disease

**DOI:** 10.1186/s13023-024-03396-3

**Published:** 2024-10-16

**Authors:** Han Wang, Ying Yang, Lin Liu, Yawen Zhao, Yang Li, Wei Zhang, Wei Ma

**Affiliations:** 1 Department of Cardiology, Peking University First Hospital, 8 Xishiku St, Xicheng District, Beijing, 100034 China; 2 Department of Neurology, Peking University First Hospital, Beijing, China; 3 Department of Nephrology, Peking University First Hospital, Beijing, China; 4 Echocardiography Core Lab, Institute of cardiovascular Disease, Peking University First Hospital, Beijing, China

## Abstract

**Background:**

This study aimed to quantitatively evaluate the left ventricular global and regional myocardial work of patients in Fabry disease (FD) by echocardiographic pressure–strain loop (PSL) analysis.

**Results:**

The study included 48 patients with FD and 48 healthy controls matched for age and sex. According to the presence/absence of left ventricular hypertrophy (LVH), the patients with FD were divided into an LVH + group and an LVH– group. Left ventricular blood pressure was estimated noninvasively according to echocardiographic valvular events and systolic pressure in the brachial artery. Left ventricular myocardial work parameters were acquired by echocardiographic pressure–strain loop analysis.

The FD groups had a significantly lower global longitudinal strain (GLS), global work index, global work efficiency (GWE), global constructive work and higher global waste work than the control group (*P* < .05). Regional analysis showed that all segmental myocardial waste work increased and myocardial work efficiency decreased in the LVH + group than in the LVH– group (*P* < .05). Segmental longitudinal strain, myocardial work index, and myocardial constructive work were markedly lower in the basal and middle segments (*P* < .05) and preserved in the apical segments. Multivariate analysis revealed that GWE and GLS were significant related to LVH.

**Conclusions:**

Myocardial work analysis can be used to assess global and regional myocardial work in patients with FD. In this study, GLS and GWE were reduced in patients with FD and associated with the presence of LVH. Basal and middle myocardial work decreased in relation to the LVH, while apical myocardial work remained, which added value to explore the distribution of myocardial impairment.

## Introduction

Fabry disease (FD, OMIM 301500) is an X-linked chromosomal disorder caused by a pathogenic variant in the GLA gene, which leads to decrease the activity of α-galactosidase A (α-Gal A) and progressive accumulation of complex sphingolipids, predominantly globotriaosylceramide (Gb3) and lyso-globotriaosylceramide (lyso-Gb3) [1]. Gb3 and lyso-Gb3 can deposit in many types of parenchymal cells, leading to the dysfunction of multiple organs [2]. Cardiac involvement is common in FD and manifests morphologically as concentric remodeling or left ventricular hypertrophy (LVH) with consequent alterations in the left ventricular (LV) myocardium [3, 4]. Previous studies have shown that FD-related cardiac disease is the leading cause of mortality, accounting for 75% of all deaths, and that LV ejection fraction (LVEF) in the majority of patients with FD remains within the normal range until the later stages in the majority of patients with the disease [5, 6].

Speckle-tracking echocardiography (STE) was developed to calculate the speed, displacement of selected region to calculate myocardial tissue motion, with less dependence on geometric assumptions and loading conditions than conventional echocardiographic parameters [7]. Left ventricular global longitudinal strain (LVGLS) has been reported to be more sensitive than LVEF for early detection of minor myocardial damage and has proven benefit in terms of both diagnosis and risk stratification in FD patients with FD [5], but is limited by loading conditions. On the basis of STE, myocardial work is a novel non-invasive method to characterize systolic myocardial deformation in relation to afterload conditions, and is measured at both the segmental and global levels [8], which may be prior to strain in identifying the early abnormalities of LV systolic function and establishing a more sensitive index for early detection of myocardial dysfunction in patients with FD [9].

In this study, we observed the myocardial work by echocardiographic pressure–strain loop analysis in patients with FD with the aims of detecting functional myocardial alterations and determining the contribution of segmental myocardial work to global myocardial work.

## Methods

### Study population

We retrospectively screened the cohort of 65 patients with FD, who underwent echocardiography in Peking University First Hospital, China, from August 2014 to January 2023 in accordance with the 2021 Chinese consensus [10]. Seventeen patients who were under 18 years of age or had echocardiographic images of insufficient quality were excluded, leaving 48 adult patients eligible for measurement of myocardial work Patients were divided in two groups according to maximal LV wall thickness: patients with LVH (defined as LV thickness ≥ 12 mm, LVH +) and without LVH (LV wall thickness < 12 mm, LVH-) [4, 11]. The control group consisted of 48 healthy subjects who were defined as all those individuals with absence of any disease and cardiovascular risk factors, matched for age and gender.

### Definition of fabry disease

FD was diagnosed based on the typical clinical symptoms, family history, and measurement of the enzymatic activity of α-galactosidase A in dried blood spots (DBS), plasma Lyso-Gb3 and genetic testing to demonstrate the mutation in the GLA gene in accordance with the 2021 Chinese consensus statement [10]. The study protocol was conducted in accordance with the principles of the Declaration of Helsinki and the ethical standards of the relevant national and institutional committees on human experimentation and approved by the Institutional Review Board of Peking University First Hospital [No. 2023 (527–001)].

### Echocardiographic analysis

#### Conventional echocardiographic study

Before the examination, brachial artery systolic pressure was measured. Echocardiography was performed by an experienced sonographer in accordance with American Society Echocardiography guidelines [12], using the Vivid E95 ultrasound system (GE Healthcare, America) with an M5S transducer. All images were recorded in DICOM format and transmitted to an external hard disk.

LVEF was obtained by the Simpson’s biplane method. LV end-diastolic and end-systolic dimensions and LV wall thickness were obtained from two-dimensional or M-mode images. LV end-diastolic and end-systolic volumes were measured from the apical 2-chamber and 4-chamber views. The LV mass (LVM) was calculated from formula. Parameters were normalized to body surface area (BSA).

#### Longitudinal strain and myocardial work analysis

**Fig. 1:**
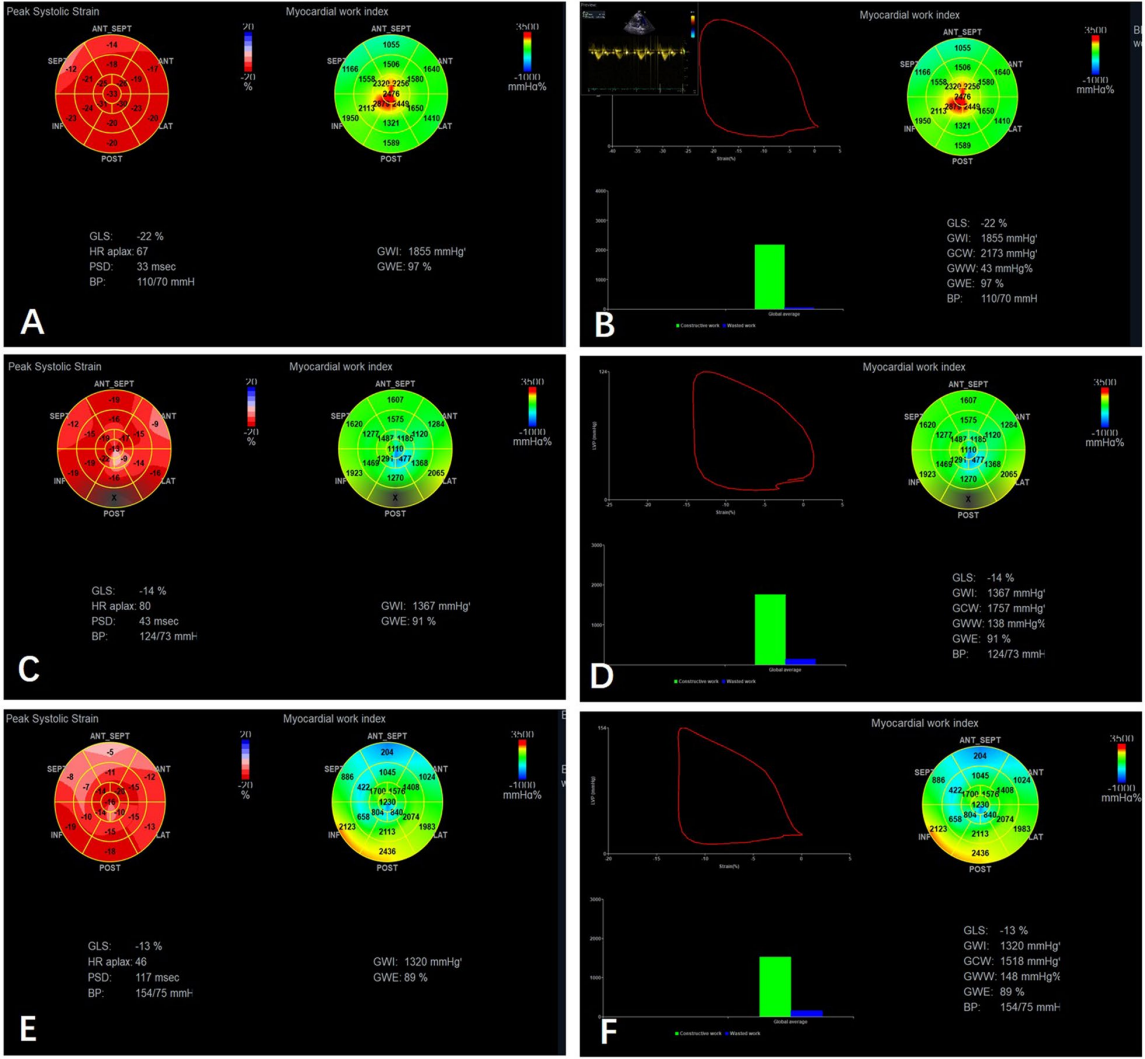
Left ventricular longitudinal strain and myocardial work. Examples of patients from: (**A, B**) Control, (**C, D**) FD group without LVH, (**E, F**) FD group with LVH. Each graph shows the bull’s eye map of segmental longitudinal strain and myocardial work index (left); the noninvasive pressure-strain loop, global constructive work, global wasted work and segmental myocardial work index (right)

Speckle-tracking was performed on the apical 4-chamber, 3-chamber and 2-chamber views, and LVGLS was calculated by averaging the peak value of three apical views [13]. The bull's eye diagram of LVGLS was constructed and then a pressure–strain loop (PSL) was obtained by fitting the individualized reference curve in accordance with the principles for estimation of LV pressure and work [8]. The areas of the pressure–strain loop represented the global myocardial work index (GWI), the myocardial work performed during segmental shortening is represented as global constructive work (GCW), and myocardial work performed during segmental elongation represented as global waste work (GWW). The global work efficiency (GWE) is the ratio of GCW to (GCW + GWW). All Strain measurements were analyzed by the EchoPAC software (version 204, GE Vingmed Ultrasound, Norway), shown in Fig. 1. The myocardium border detection was automated to ensure optimal tracking throughout the cardiac cycle by the software, and the operator conformed that the true boundaries were traced.

The ventricle was divided into the following 18 segments for exploration of the distribution of regional strain and work in the left ventricle: basal inferior (S1), basal inferior (S2), basal lateral (S3), basal anterior (S4), basal anteroseptal (S5), and basal septal (S6); middle inferior (S7), middle inferior (S8), middle lateral (S9), middle anterior (S10), middle anteroseptal (S11), and middle septal (S12); apical inferior (S13), apical inferior (S14), apical lateral (S15), apical anterior (S16), apical anteroseptal (S17), and apical septal (S18). The parameters were subsequently calculated as the average value of each of the 6 segments: longitudinal strain (LS) of basal segments, middle segments, apical segments (Bas-LS, Mid-LS, Api-LS); myocardial work index of basal segments, middle segments, apical segments (Bas-MWI, Mid-MWI, Api-MWI); myocardial constructive work of basal segments, middle segments, apical segments (Bas-MCW, Mid-MCW, Api-MCW); myocardial waste work of basal segments, middle segments, apical segments (Bas-MWW, Mid-MWW, Api-MWW); myocardial work efficiency of basal segments, middle segments, and apical segments (Bas-MWE, Mid-MWE, Api-MWE).

The reproducibility in measurement of myocardial work was evaluated in 10 randomly selected patients using the interclass correlation coefficient, and the results are shown in Appendix 1.

### Statistical analysis

Statistical calculations were performed using IBM SPSS Statistics version 23.0 (IBM SPSS Statistics, IBM Corporation, Armonk, New York). Continuous variables were assessed for normality of distribution using the Kolmogorov–Smirnov tests and are expressed as the mean ± SD. Categorical data are reported as the counts and percentages. Comparisons between two groups used *t*-tests for continuous variables, and the chi-squared test for categorical data. Differences among groups (the control, FD, LVH +, and LVH- groups) of continuous variables were assessed by analysis of variance, and chi-squared test was applied to compare dichotomous variables. Variables associated with changes in LVH in the univariate analysis (*P* < 0.05) were included in the multivariate analysis, after adjustment for age. The Bland–Altman method was used to assess the consistency of myocardial work parameters between and within observers. Two-sided *P* < 0.05 was considered statistically significant.

## Results

### Characteristics of patients with FD

Table 1 presents the demographic and clinical data for the patients with FD and the controls, and there was no significant difference in sex or age. However, there were statistically significant differences in the estimated glomerular filtration rate (eGFR), serum creatinine, and brain natriuretic peptide between the two groups (*P* < 0.05).

**Table 1:** Clinical characteristics and echocardiographic data in FD patients and healthy subjects

Variable	Control group	FD group	*P*
	(*n* = 48)	(*n* = 48)	
Age, yrs	39.1 ± 14.4	39.1 ± 14.4	–
Onset-age, yrs	–	12.36 (7.00, 10.00)	–
Interval year, yrs	–	23.50 (12.50, 33.75)	–
Female (n, %)	18 (37.5)	18 (37.5)	–
BMI, kg/m^2^	23.6 ± 3.4	22.1 ± 4.3	0.057
BSA, m^2^	1.7 ± 0.2	1.7 ± 0.2	0.352
Systolic BP, mm Hg	124.4 ± 7.9	126.8 ± 19.0	0.424
Diastolic BP, mmHg	76.9 ± 7.4	74.6 ± 18.1	0.407
Basic heart rate, beats/min	77.2 ± 7.7	74.7 ± 15.7	0.336
Hypertension (n, %)	–	18 (52.9)	< 0.001
Diabetes mellitus (n, %)	–	1 (2.9)	0.415
Hyperlipidemia (n, %)	–	5 (22.7)	0.002
SCR, μmol/L	73.0 ± 12.0	216.3 ± 359.8	0.007
eGFR, mL/ min/1.73m^2^	104.6 ± 17.0	74.0 ± 42.1	< 0.001
UA, μmol/L	342.9 ± 75.7	369.8 ± 110.4	0.243
BNP, pg/ml	22.4 ± 18.9	595.2 ± 981.2	< 0.001
α-Gal A	–	3.47 (0.35–1.95)	–
Lyso-Gb3	–	50.88 (10.43–86.24)	–
**Echocardiographic measurements**			
LVEF, %	73.4 ± 3.8	70.3 ± 8.9	0.027
iLVEDd, cm/m^2^	2.6 ± 0.2	2.7 ± 0.3	0.117
iLVESd, cm/m^2^	1.5 ± 0.2	1.6 ± 0.3	0.028
iLVEDV, ml/m^2^	52.2 ± 10.3	55.5 ± 13.4	0.184
iLVESV, ml/m^2^	13.8 ± 3.3	17.0 ± 8.9	0.022
IVSd, cm	0.9 ± 0.1	1.2 ± 0.5	< 0.001
LVPW, cm	0.9 ± 0.1	1.2 ± 0.4	< 0.001
LVMI, g/m^2^	77.3 ± 16.5	124.7 ± 72.1	< 0.001

FD: Fabry disease; BMI: body mass index; BSA: body surface area; BP: blood pressure; SCR: serum creatinine; eGFR: estimated glomerular filtration rate (by the formula of Cockcroft-Gault); UA: uric acid; BNP: brain natriuretic peptide; α-Gal A: α-galactosidase A (reference interval 2.20–17.65 μmol/L/h); Lyso-Gb3: globotriaosylsphingosine (reference interval < 1.11 ng/mL); LVEF: left ventricular ejection fraction; iLVEDd: indexed LV end-diastolic dimension; iLVESd: indexed LV end-systolic dimension; iLVEDV: indexed LV end-diastolic volume; iLVESV: indexed LV end-systolic volume; IVSd: interventricular septum thickness; LVPW: LV posterior wall thickness; LVMI: indexed LV mass

The mean age of the patients with FD was 39.1 ± 14.4 years and 62.5% were male. There were 33 patients in the LVH—group and 15 in the LVH + group (Table 2**)**. There was no significant difference in disease onset age between two groups. In the LVH + group, the interval between disease onset and transthoracic echocardiographic examination was 39.0 (37.3–47.3) years, which was longer than that in the LVH – group. Thirty-six patients underwent α-Gal A activity measurement and the median value was 3.5 μmol/L/h (interquartile range [IQR 0.4, 2.0]). Twenty-nine patients underwent plasma Lyso-Gb3 measurement and the median value was 50.9 ng/mL (IQR 10.4, 86.2). Significant differences were found in eGFR, serum creatinine, uric acid, and brain natriuretic peptide. Systolic blood pressure was higher in the LVH + group, with a diagnosis of hypertension being more common in the LVH + group than in the LVH– group (*n* = 11 [78.6%] vs *n* = 7 [35.0%]).

**Table 2:** Comparative analysis in FD patients with or without LVH

Variable	LVH—group	LVH + group	*P*
	(n = 33)	(n = 15)	
Age, yrs	33.21 ± 11.58	52.07 ± 11.23	< 0.001
Onset-age, yrs	12.83 (7.00–10.00)	10.25 (8.00–10.75)	0.672
Interval year, yrs	20.06 (11.00–25.75)	39.00 (37.25–47.25)	0.014
Female (n, %)	12 (36.36%)	6 (40.00%)	0.809
BMI, kg/m^2^	22.11 ± 4.55	22.10 ± 3.86	0.995
BSA, m^2^	1.68 ± 0.18	1.63 ± 0.20	0.457
Systolic BP, mm Hg	121.48 ± 12.55	137.00 ± 24.91	0.008
Diastolic BP, mmHg	73.28 ± 9.44	77.07 ± 28.65	0.517
Basic heart rate, beats/min	75.90 ± 17.47	73.00 ± 13.34	0.605
Hypertension (n, %)	7 (35.00%)	11 (78.57%)	0.012
Diabetes mellitus (n, %)	1 (5.00%)	0 (0.00%)	0.396
Hyperlipidemia (n, %)	2 (16.67%)	3 (30.00%)	0.457
SCR, μmol/L	85.38 ± 30.98	437.99 ± 29.59	0.003
eGFR, mL/ min/1.73m^2^	97.88 ± 28.73	42.79 ± 36.40	< 0.001
UA, μmol/L	323.58 ± 100.67	431.44 ± 95.23	0.022
BNP, pg/ml	56.60 ± 101.19	1044.08 ± 1160.19	0.015
α-Gal A	3.83 (0.25–2.40)	2.71(0.35–1.22)	0.644
Lyso-Gb3	59.81(10.74–88.34)	31.05 (9.58–46.40)	0.121
LVEF, %	71.83 ± 6.38	66.87 ± 12.40	0.072
iLVEDd, cm/m^2^	2.67 ± 0.32	2.72 ± 0.22	0.567
iLVESd, cm/m^2^	1.57 ± 0.26	1.69 ± 0.32	0.189
iLVEDV, ml/m^2^	54.81 ± 13.73	56.87 ± 13.07	0.628
iLVESV, ml/m^2^	15.70 ± 6.08	19.98 ± 12.93	0.123
IVSd, cm	0.98 ± 0.14	1.81 ± 0.39	< 0.001
LVPW, cm	0.97 ± 0.18	1.55 ± 0.39	< 0.001
LVMI, g/m^2^	88.19 ± 21.73	204.97 ± 79.61	< 0.001

FD: Fabry disease; BMI: body mass index; BSA: body surface area; BP: blood pressure; SCR: serum creatinine; eGFR: estimated glomerular filtration rate (by the formula of Cockcroft-Gault); UA: uric acid; BNP: brain natriuretic peptide; α-Gal A: α-galactosidase A (reference interval 2.20–17.65 μmol/L/h); Lyso-Gb3: globotriaosylsphingosine (reference interval < 1.11 ng/mL); LVEF: left ventricular ejection fraction; iLVEDd: indexed LV end-diastolic dimension; iLVESd: indexed LV end-systolic dimension; iLVEDV: indexed LV end-diastolic volume; iLVESV: indexed LV end-systolic volume; IVSd: interventricular septum thickness; LVPW: LV posterior wall thickness; LVMI: indexed LV mass

### Echocardiographic parameters

Mean LVEF was 70.3 ± 8.9% in the FD group and lower than the control group, shown in the Table 1. Interventricular septum and LV posterior wall thickness were signficantly higher in the patients with FD than controls (*P* < 0.01), as were LV mass, end-systolic dimension, and end-systolic volume (*P* < 0.05).

Table 2 summarizes the main echocardiographic findings in the LVH + and LVH- groups. There were no differences between the two groups in LVEF or LV dimensions and volumes with the exception of LV mass (205.0 ± 79.6 g/m^2^ vs 88.2 ± 21.7 g/m^2^, *P* < 0.01).

### GLS and left ventricular myocardial work

Comparison of patients with FD and controls.

**Fig. 2:**
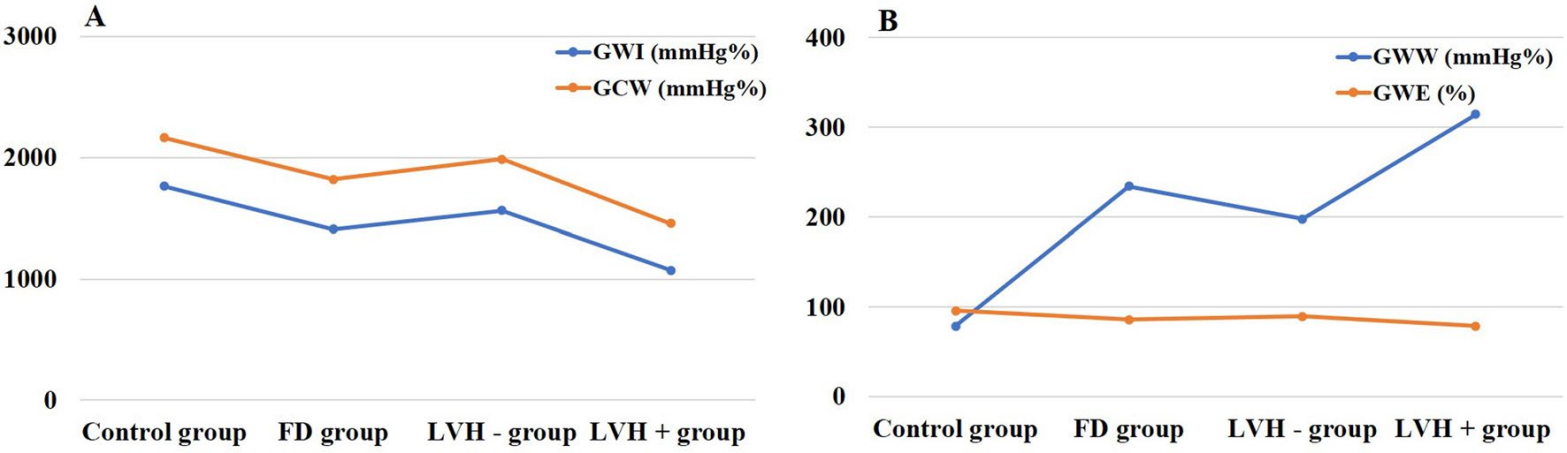
Myocardial work in FD patients with or without LVH and control subjects. **A** GWI and GCW, **B** GWW and GWE. FD, Fabry disease; LVH, left ventricular hypertrophy; GWI global work index; GCW, global constructive work; GWW, global waste work; GWE, global work efficiency

The results for STE and myocardial work parameters are summarized in Tables 3. The GLS, GWI, GCW, and GWE were lower and GWW was higher in patients with FD than in controls (*P* < 0.01), shown in Fig. 2.

**Table 3:** GLS and myocardial work parameters

Variable	Control group	FD group	LVH—group	LVH + group	*p*
	(n = 48)	(n = 48)	(n = 33)	(n = 15)	
GLS (%)	−21.29 ± 2.10	−15.26 ± 4.77a	−17.05 ± 3.78a	−11.33 ± 4.43abc	< 0.001
GWI (mmHg%)	1764.19 ± 230.57	1409.88 ± 480.92a	1563.73 ± 401.80	1071.40 ± 478.44abc	< 0.001
GWE (%)	95.56 ± 1.58	85.81 ± 8.83a	89.12 ± 6.87a	78.53 ± 8.46abc	< 0.001
GCW (mmHg%)	2162.12 ± 244.58	1820.46 ± 505.47a	1986.18 ± 405.71	1455.87 ± 523.78abc	< 0.001
GWW (mmHg%)	78.42 ± 37.47	234.04 ± 148.01a	197.42 ± 124.16a	314.60 ± 167.92ac	< 0.001

FD: Fabry disease; GLS: global longitudinal strain; GWI: global myocardial work index; GWE: global myocardial work efficiency; GCW: global myocardial constructive work; GWW: global myocardial waste work

*P* < 0.05 versus control group, ^b^ *P* < 0.05 versus FD group, ^c^ *P* < 0.05 versus LVH- group

Comparison of LVH + and LVH– groups.

GLS was signficantly impaired in the LVH + group in comparison with the LVH- group and the control group (*P* < 0.01). Similarly, GWI, GWE and GCW were lower and GWW was higher in the LVH + group than in the LVH- and control group (*P* < 0.01). Of note, there was no significant difference in GWI or GCW between the LVH- group and the control group; however, GLS and GWE were worse and GWW was increased in the LVH– group (*P* < 0.01) (Fig. 2**)**.

### Correlation analysis of GLS and myocardial work parameters with LVH

Correlation analysis revealed a good correlation of GLS and myocardial work parameters with LVH respectively. Multivariate analysis showed that GLS and MWE were associated with LVH (Table 4).

**Table 4:** Univariate and multivariate analysis for FD patients with LVH

Variable	Univariate analysis		Multivariate analysis	
	OR (95% CI)	*P*	OR (95% CI)	*P*
Age, yrs	1.15 (1.06, 1.25)	0.0007		
GLS (%)	1.45 (1.15, 1.83)	0.0015	1.29 (1.01, 1.64)	0.0402
GWI (mmHg%)	1.00 (1.00, 1.00)	0.003	1.00 (1.00, 1.00)	0.1291
GWE (%)	0.83 (0.74, 0.94)	0.0019	0.87 (0.77, 0.98)	0.0244
GCW (mmHg%)	1.00 (1.00, 1.00)	0.0021	1.00 (1.00, 1.00)	0.1839
GWW (mmHg%)	1.01 (1.00, 1.01)	0.0191	1.00 (1.00, 1.01)	0.1527

The multivariate analysis was adjusted by age and only variables that were significant in the multivariate analysis are shown.

### Regional left ventricular strain and myocardial work

Compared with those in the LVH – group, Bas-LS and Mid-LS were reduced significantly in the LVH + group (*P* < 0.05), while Api-LS was preserved (*P* = 0.40). MWE was lower and MWW was higher in all segments in the LVH + group than in the LVH- group (*P* < 0.05). Additionally, MWI and MCW showed a decreasing trend, within an obvious significant in basal and middle segments in the LVH + group compared with the LVH – group, while apical MWI and MCW were preserved as shown in Table 5.

**Table 5:** Reginal analysis of longitudinal strain and myocardial work parameters

Variable	FD group	LVH—group	LVH + group	*P*
	(n = 48)	(n = 33)	(n = 15)	
Bas-LS (%)	−11.76 ± 5.84	−13.95 ± 4.86	−6.94 ± 4.94ab	< 0.001
Mid-LS (%)	−13.64 ± 5.51	−16.44 ± 3.47a	−7.48 ± 3.92ab	< 0.001
Api-LS (%)	−17.77 ± 8.32	−18.86 ± 7.44	−15.37 ± 9.84	0.403
Bas-MWI (mmHg%)	1318.21 ± 463.91	1525.35 ± 289.68	859.52 ± 453.72ab	< 0.001
Mid-MWI (mmHg%)	1384.90 ± 391.58	1563.54 ± 271.21	989.35 ± 323.47ab	< 0.001
Api-MWI (mmHg%)	1762.85 ± 728.71	1735.44 ± 533.82	1823.55 ± 1064.86	0.933
Bas-MWE (%)	83.93 ± 11.16	88.91 ± 5.41	72.88 ± 12.77ab	< 0.001
Mid-MWE (%)	89.53 ± 6.41	92.82 ± 2.82a	82.25 ± 6.15ab	< 0.001
Api-MWE (%)	89.99 ± 7.93	91.83 ± 6.69	85.89 ± 9.12b	0.063
Bas-MCW (mmHg%)	1633.96 ± 427.32	1807.32 ± 318.53	1250.07 ± 391.82ab	< 0.001
Mid-MCW (mmHg%)	1742.27 ± 517.62	1968.06 ± 381.57	1242.31 ± 424.35ab	< 0.001
Api-MCW (mmHg%)	2219.90 ± 877.02	2196.93 ± 673.04	2270.75 ± 1246.57	0.967
Bas-MWW (mmHg%)	250.18 ± 176.37	188.42 ± 87.49	386.93 ± 241.61ab	0.001
Mid-MWW (mmHg%)	159.09 ± 75.61	135.15 ± 64.38	212.11 ± 73.42ab	0.005
Api-MWW (mmHg%)	172.13 ± 110.01	136.95 ± 86.82	250.05 ± 118.65ab	0.005

FD: Fabry disease; LS: longitudinal strain; MWI: myocardial work index; MWE: myocardial work efficiency; MCW: myocardial constructive work; MWW: myocardial waste work

^a^ *P* < 0.05 versus FD group, ^b^ *P* < 0.05 versus LVH- group

### Intraobserver and interobserver reproducibility assessment

The intraclass correlation coefficient were calculated for interobserver and intraobserver agreement in 15 randomly selected patients. Myocardial work parameters were re-measured from the same images by two independent observers who were blinded.

## Discussion

This study used strain measurements and myocardial work to observe patients with FD and healthy controls. The serum creatinine and brain natriuretic peptide were significantly higher, and eGFR, LVEF, and GLS were lower in with FD than in controls. LVH was present in 31.25%, and the interval between disease onset and echocardiographic examination was longer than the pre-hypertrophic phase of FD. We also confirmed that LVEF was relatively lower and GLS was significantly lower in the LVH + group, which is consistent with previous reports. Importantly, global and segmental myocardial work could be evaluated non-invasively by pressure–strain analysis. GWI, GWE, and GCW were lower and GWW was higher in the LVH + group than in the LVH- group and the control group. Moreover, GLS and GWE were associated with LVH in patients with FD. Comparison of segmental strain and myocardial work between the LVH + and LVH– groups revealed the following: basal and middle LS values were reduced and apical LS was preserved; basal and middle MWI and MCW were decreased but apical MWI and MCW were not; MWE was decreased and MWW was increased in all three myocardial regions.

### Global strain and myocardial work patterns in FD

Cardiac involvement in FD has been characterized by a dficient enzymatic activity of α-galactosidase A and accumulation of globotriaosylceramide (Gb3) [2, 3]. LVH is the main feature of cardiac involvement, with increased ventricular wall thickness mimicing hypertrophic cardiomyopathy [1], and this study showed that 31.25% of our FD cohort had LVH. Ventricular function measured by LVEF was lower in our FD group than in our control group. STE can distinguish abnormal myocardial deformation and detect subclinical LV dysfunction, and its role of STE in FD has already been demonstrated [5]. Shanks et al. demonstrated that two-dimensional GLS was lower in patients with FD than in controls [6]. Consistent with those studies, we observed that GLS was lower in patients with FD and in those without LVH than in controls, which suggests GLS may play a role in detection of subtle cardiac dysfunction in the pre-hypertrophic stage of FD. These findings are in agreement with previous research using STE in patients with hypertrophic cardiomyopathy [14] and other studies that have found a strong association between functional alterations in the LV myocardium and the functional class [15, 16].

Myocardial work is a novel non-invasive method to characterize myocardial deformation in relation to afterload conditions [8]. Clemmensen et al. demonstrated that the prognostic value of myocardial work was superior to GLS for prediction of adverse cardiac events and all-cause mortality in patients with cardiac amyloidosis [17]. Similarly, Spinelli et al. looked at 96 patients with Fabry disease and found all MW indices appear to be good predictors of poor cardiac outcome [18]. We also observed that FD patients had lower GLS, GWI, GCW, GWE and elevated GWW compared to healthy controls, consistent with previous reports [17–19]. Furthermore, in our study, GLS, GWI, GCW, and GWE were more severely affected in the advanced stages of FD with cardiac involvement. Experimental and clinical studies have demonstrated that myocardial work takes in account deformation as well as afterload and correlates strongly with the equivalent invasive work measurement. Chronic intracellular accumulation of glycosphingolipids leads to inflammation, hypertrophy, and interstitial fibrosis, and myocardial dysfunction, which was reflected in reduced GLS and MWI in our study. As LV remodeling proceeds, decreased GLS and increased GWW lead to a poor GWE, which is likely related to the increased ventricular stiffness caused by myocardial impairment. We had shown that GLS and GWE correlates negatively with LVH, suggesting myocardial work might reflect the stage and severity of overt cardiomyopathy in FD.

### Segmental strain and myocardial work patterns in FD

Cardiac involvement is common in FD and morphologically manifests as concentric remodeling or hypertrophy of the left ventricle, with replacement fibrosis usually located in the posterolateral wall [1]. Weidemann et al. found that late gadolinium enhancement cardiac magnetic resonance images demonstrated a particular distribution of late gadolinium enhancement to the infero-lateral wall of the middle to basal LV and to the mid-myocardial layer in patients with FD [20]. In our patients, basal and middle LS were reduced in the LVH + group, while apical LS was similar between the two groups. Myocardial work may be superior to myocardial strain when assessed by noninvasive LV pressure–longitudinal strain curves. We observed that MWI, MCW, and MWE in the basal and middle segments decreased in relation to the LVH, with an accompanying increase in segmental MWW. Interestingly, the present study showed that apical MWE decreased and apical MWW increased, while apical MWI and MCW were preserved, which implies that the apical myocardial properties had already been affected, despite systolic function being apparently preserved. Previous studies have shown basal regions had a greater radius of curvature compared to the apical regions, leading to an increased exposure to wall stress as posited by the Laplace’s Law [21–23]. As a consequence of this development, the imbalance between dramatically elevated wall stress and locally developed force resulted in decreased local deformation [24]. The preserved function in the apical region could be helpful to maintain normal global deformation, thereby compensating for the impairment in the basal region. The stages of cardiac involvement in patients with FD, particularly LVH, may trigger regional myocardial impairment and break this severe imbalance, leading to a further increase in MWW across all apical segments and damage to the apical myocardia properties.

## Study limitations

Several limitations should be mentioned. First, it had a relatively small sample size. Second, our cohort was from the same center, which may affect the generalizability of its findings. Third, this study excluded patients with poor quality images, which might cause selection bias. Therefore, a multicenter study in a larger sample size is needed to confirm our findings.

## Conclusion

Myocardial work analysis based on noninvasive LV pressure–longitudinal strain curves is feasible for most patients with FD. GLS and GWE correlate strongly with in Fabry patients with cardiac involvement according to the presence of LVH. Myocardial work in basal and middle segments decreased in relation to the LVH, while apical myocardial work remained basically preserved, suggesting that the equilibrium of apical systolic properties is preserved in both the pre-hypertrophic stage and hypertrophic stage. Myocardial work parameters could be useful tools for more dynamic exploration of the distribution of global and segmental myocardial impairment in FD.

## Data Availability

The authors confirm that the data supporting the findings of this study are available within the article and its supplementary material.

## Appendix 1

See Table

**Table 6:** Interclass correlation coefficients for strain measurements and myocardial work parameters

Variable	Interobserver		Intraobserver	
	ICC	P	ICC	P
*LVGLS*	*0.952*	< *.001*	*0.982*	< *.001*
*GWI*	*0.946*	< *.001*	*0.973*	< *.001*
*GWE*	*0.931*	< *.001*	*0.961*	< *.001*
*GCW*	*0.933*	< *.001*	*0.934*	< *.001*
*GWW*	*0.899*	< *.001*	*0.921*	< *.001*

ICC: intraclass correlation coefficient; GLS: global longitudinal strain; GWI: global myocardial work index; GWE: global myocardial work efficiency; GCW: global myocardial constructive work; GWW: global myocardial waste work

6
